# Does the Keele STarT Back Screening Tool Contribute to Effectiveness in Treatment and Cost and Loss of Follow-Up of the Mechanical Diagnosis and Therapy for Patients with Low Back Pain?

**DOI:** 10.3390/diagnostics10080536

**Published:** 2020-07-30

**Authors:** Takahiro Tsuge, Hiroshi Takasaki, Michio Toda

**Affiliations:** 1 Department of Rehabilitation, Kurashiki Medical Center, 250 Bakuro, Kurashiki, Okayama 710-8522, Japan; takahiro_tsuge@yahoo.co.jp; 2 Department of Physical Therapy, Saitama Prefectural University, Koshigaya, Saitama 343-8540, Japan; 3 Department of Orthopaedic Surgery, Kurashiki Medical Center, 250 Bakuro, Kurashiki, Okayama 710-8522, Japan; todagano@yahoo.co.jp

**Keywords:** classification, discharge, follow-up, Keele STarT Back Screening Tool, McKenzie, mechanical diagnosis and therapy, stratified model of care, subgroup

## Abstract

Background: Mechanical diagnosis and therapy (MDT) and the stratified approach using the Keele STarT Back Screening Tool (SBST) are examples of stratified low back pain (LBP) management. We investigated whether the medium–high risk in SBST can contribute to the time and sessions until discharge from MDT (Question 1) and to the loss of follow-up before identifying a promising management strategy (Question 2). Methods: A retrospective chart study was conducted. Multiple regression modeling was constructed using 10 independent variables, including whether the SBST was medium–high risk or not for Question 1, and the 9/10 independent variables for Question 2. Results: The data of 89 participants for Question 1 and 166 participants for Question 2 were analyzed. SBST was not a primary contributing factor for Question 1 (*R*^2^ = 0.17–0.19). The model for Question 2 included SBST as a primary contributing factor and the shortest distance from the patient address to the hospital as a secondary contributing factor (93.4% correct classification). Conclusion: SBST status was not a primary contributing factor for time and sessions until discharge from MDT, but was a critical factor for the loss of MDT follow-up before identifying a promising management strategy.

## 1. Introduction

A stratified care model is known as a promising management strategy for low back pain (LBP) [1]. In this care model, patients are categorized into subgroups, and certain interventions that respond to a specific subgroup are provided. The subgroups can be created based on key characteristics such as symptom responses to examinations and/or their prognostic profile. Over the last two decades, identifying the subgroups and targeted interventions has become a research hotspot [2,3].

One example of the stratified care model is the McKenzie method of the mechanical diagnosis and therapy (MDT), which has been effective in both treatment and cost for managing LBP [4,5,6,7], and is the most widely used physical therapy management for LBP [8,9,10,11]. MDT focuses on patient education and the empowerment of the patient’s self-management [12]. MDT therapists have more biopsychosocial perspectives and greater compliance with clinical practice guidelines for the management of LBP than other physical therapists [13]. MDT management strategies are individualized by considering biopsychosocial perspectives, which may correct abnormal cognitive processing for pain and psychological status that disturbs recovery from LBP (yellow-frags) [14,15]. In addition, the effectiveness of MDT in the population with chronic LBP has been established in a recent systematic review [16], who are likely to have some yellow-frags [17,18]. In MDT, there are three primary subgroups for the spinal problem, and the inter-examiner reliability for the subgroups has been established among credentialed MDT therapists [19]. The most prevalent subgroup is for derangement syndrome [20,21,22,23,24]. The derangement syndrome subgroup has a specific direction of loading, resulting in functional improvement and pain, which is called a directional preference (DP). The use of mechanical loading toward the DP is strongly recommended in clinical practice guidelines for LBP [25].

Another example of the stratified care model is to select management strategies based on a patient-reported outcome measure (PROM), the Keele STarT Back Screening Tool (SBST) [26]. SBST is a screening tool to help clinicians estimate the risk of persistent disabling symptoms, and the medium-high-risk group (SBST total score ≥ 4) has a poorer prognosis than the low-risk group (SBST total score < 4) [27]. The treatment effect increases when the physical therapists provide management with information about SBST [28]. Such a screening tool that comprises question items associated with yellow-flags is generally accepted by the MDT therapists in order to enhance the quality of the evaluation and treatment [29], and may be useful for MDT therapists as it may be difficult to accurately understand a patient’s psychological status through physical examinations [30]. Considering the two stratified care models, it has been questioned whether the medium-high-risk group in SBST is a contributing factor for the treatment effect of MDT, which is reflected by time until discharge from MDT. It has also been speculated whether the medium-high-risk group in SBST is a contributing factor for the cost taken until discharge, which is reflected by the number of sessions until discharge from MDT.

When time and sessions until discharge from the MDT are considered, it is also important to remember that there are some patients who lose follow-ups before identifying effective management strategies. We have known that a long duration of LBP and many comorbidities [21,22] are contributing factors for the loss of MDT follow-up; however, no study has investigated whether the medium-high-risk group in SBST can also be included as a contributing factor. In the MDT for the spinal problem, the subgroup was identified within five sessions [31]. Those who could not identify a subgroup were categorized into the mechanically inconclusive subgroup [31]. In MDT, it is impossible to identify effective management strategies for the mechanically inconclusive subgroup, and thus it is important to advise them to consult other professionals. However, there is no useful information about considering a further management strategy when the patient in the provisional subgroup of mechanically inconclusive loses the follow-up before the fifth session, resulting in a loss of time and effort for the patient and therapist.

This study investigated the following two questions:

Question 1: Whether the medium-high-risk group in SBST can be a contributing factor for time and the number of sessions until discharge from MDT?

Question 2: Whether the medium-high-risk group in SBST can be a contributing factor for the loss of follow-up with the mechanically inconclusive subgroup before the fifth session?

## 2. Materials and Methods

### 2.1. Design

A retrospective chart study was conducted. Participants’ identifying information were not used in this study, and this study was granted by an institutional research ethics committee (Kurashiki Medical Center, No. 716, 12 November 2019).

### 2.2. Participants

Prerecorded medical records in the Kurashiki Medical Center from 1 December 2016 to 15 October 2019 were reviewed. The inclusion criteria of the participants were as follows: (1) out-patients with a primary symptom of LBP; (2) those who were referred to physical therapy in the Kurashiki Medical Center for the conservative management of LBP, where MDT is routinely performed; and (3) those who completed PROMs at the initial physical therapy session. The exclusion criteria of the participants were as follows: (1) those who lost MDT follow-up with a clear reason for needing other medical care (e.g., fracture, surgery, and hospitalization due to other medical conditions) and (2) those with missing dependent variables. For Question 1, those who did not complete MDT until discharge were also excluded.

### 2.3. Exposure

MDT was performed by an author (T.T.) who was a credentialed MDT therapist. The MDT subgroup was assessed and the management strategies that corresponded to the MDT subgroups were given [31]. The MDT subgroup was confirmed every session (20–40 min) until a final MDT subgroup was determined, after which there were no more changes in the subgroup [32]. Interventions were individualized at each session using the guide by the MDT subgroup, and the date of the next session was decided by each patient.

The MDT therapist (34 years, male, and the final academic degree of the Bachelor degree) had 12 years of clinical experience and 8 years of MDT experience after receiving certification as a credentialed MDT therapist. Regarding the characteristics of the MDT therapist, the Japanese Adherence to Low Back Pain Practice Guidelines [13] indicated 100% guideline adherence. The biomedical/behavioral ratio in the Japanese Pain Attitudes and Beliefs Scale for Physiotherapists [13] was 0.6, where a value greater than 1 indicates more weight on biomedical treatment than biopsychosocial treatment, and a smaller value than 1 indicates more weight on biopsychosocial treatment than biomedical treatment. The Healthcare Providers Patient-Activation Scale (20--100) [33] was 69, where a smaller value indicates a therapist’s behavior is more geared toward the importance of patient self-management and patient-activation.

### 2.4. Outcomes

#### 2.4.1. Outcomes for Question 1

The dependent variable was the number of days until discharge from the MDT or the number of MDT sessions until discharge. The following 10 independent variables were included in this study: (1) the SBST status at the initial physical therapy session (low-risk/medium-high-risk) [34,35]; (2) whether the final MDT subgroup was the derangement syndrome subgroup or not; (3) patient age; (4) gender; (5) the shortest distance from the patient address to the hospital (km), which was evaluated by Google Maps; (6) LBP symptom area, which was assessed as the sum of the number of body parts presented in previous studies [36,37] (0–11); (7) duration of the current episode of LBP, defined as months since the last pain-free month [36,38]; (8) the number of medical diagnoses including commodities that were recorded in the clinical database in the Kurashiki Medical Center, diagnosis of other musculoskeletal disorders, and the current LBP diagnosis; (9) Japanese version of the Roland–Morris Disability Questionnaire (RMDQ; 0–24) [39]; and (10) an 11-point numerical pain rating scale on LBP at the initial physical therapy session (NRS-LBP; 0–10).

#### 2.4.2. Outcomes for Question 2

The dependent variable was whether the patient was lost to follow-up with the mechanically inconclusive subgroup before the fifth session or not. The independent variables in Question 2 included the same nine independent variables as Question 1, except whether the final MDT subgroup was the derangement syndrome subgroup or not.

### 2.5. Statistics

For the regression modeling, the variables that presented a left-skewed distribution were log-transformed. The correlation matrix was inspected using Spearman’s rank and Pearson’s correlation coefficients. The independent variables with a correlation coefficient >0.9 to the dependent variable were excluded in the modeling. For Question 1, the stepwise multiple regression analysis was conducted. The interpretation of the *R*^2^-value was as follows: <0.3, a none-very weak effect size; 0.3–0.5, a weak-low effect size; 0.5–0.7, a moderate effect size; and >0.7, a strong effect size [40]. For Question 2, the forward stepwise binary logistic regression analysis was conducted. SPSS version 21.0 (IBM Corporation, New York, NY, USA) was used for the statistical analyses, where the statistical significance was set at 5%. Descriptive statistics were used to summarize the participants’ characteristics.

## 3. Results

**Figure 1:**
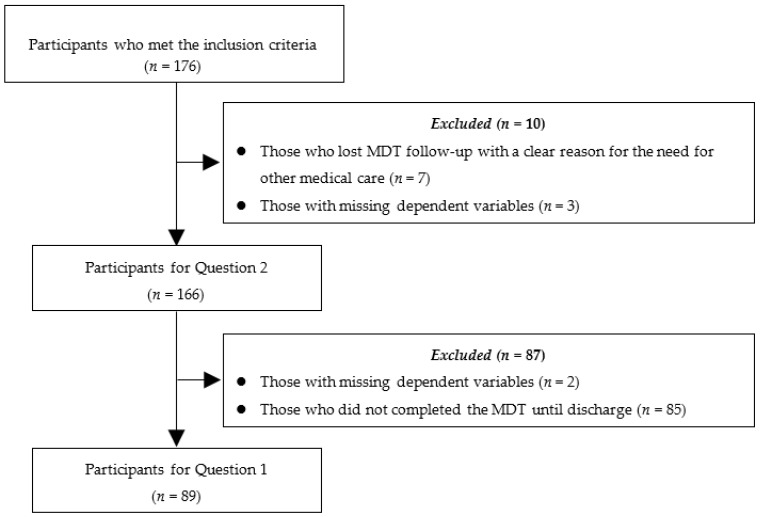
Flow of inclusion/exclusion of the participants. Abbreviations: MDT—mechanical diagnosis and therapy.

The data of 176 participants were initially included, and finally, the data of 89 participants who completed the MDT until discharge were analyzed for Question 1, and the data of 166 participants were analyzed for Question 2 (Figure 1). The characteristics of the participants are summarized in Table 1. Variables of the shortest distance from the patient address to the hospital, symptom area, the duration of the current episode of LBP, and the number of medical diagnoses were log-transformed for modeling.

For Question 1, 73 participants were in the final MDT subgroup of derangement syndrome (82.0%). Regarding SBST, 39 participants were in the low-risk group and 50 participants were in the medium-high-risk group. The mean (standard deviation (SD)) number of days until discharge from MDT was 110.0 (94.3) days, 91.0 (58.8) days in the subgroup of derangement syndrome, 197.4 (156.2) days in the subgroup of non-derangement syndrome, 101.9 (62.6) days in the low-risk group on SBST, and 116.6 (112.6) days in the medium-high-risk group on SBST. The mean (SD) number of sessions until discharge from the MDT was 7.1 (4.2) sessions, where 6.4 (3.5) sessions were in the subgroup of derangement syndrome, 10.3 (5.7) sessions in the subgroup of non-derangement syndrome, 6.0 (3.2) sessions in the low-risk group on SBST, and 7.9 (4.7) sessions in the medium-high-risk group on SBST.

For Question 2, 13 participants (7.8%) were lost to the MDT follow-up in the mechanically inconclusive subgroup before the fifth session, two patients (1.2%) were lost in the low-risk group, and 11 participants (6.6%) in the medium-high-risk group in SBST. The mean (SD) of the shortest distance from the patient address to the hospital was 5.0 (3.9) km in those that lost the MDT follow-up in the mechanically inconclusive subgroup before the fifth session, and 7.7 (6.7) km in others.

### 3.1. Question 1

There was no dependent variable with a correlation coefficient >0.9 to the dependent variable, and all 10 independent variables were included in the analysis. SBST was not a statistically significant contributing factor for the number of days until discharge from the MDT (Table 2). However, SBST was a statistically significant contributing factor for the number of sessions until discharge (Table 3). The results of the analysis of variance (ANOVA) were statistically significant for each modeling. The R^2^-values in each modeling indicated a none-very weak effect size. The Durbin–Watson statistic was 1.58 for the number of days until discharge, and 1.93 for the number of sessions until discharge. There was no outlier for which the predicted value of the measured value was above ±3 SD in each modeling.

### 3.2. Question 2

There was no dependent variable with a correlation coefficient >0.9 to the dependent variable, and all of the nine independent variables were included in the modeling. The mediumhigh-risk group in SBST was 4.61 times more likely to lose MDT follow-up with the mechanically inconclusive subgroup before the fifth session than the low-risk group in SBST (Table 4). The shortest distance from the patient address to the hospital was negatively correlated with the loss of MDT follow-up with the mechanically inconclusive subgroup before the fifth session. For the goodness of fit, the Pearson chi-square test was statistically significant (*p* < 0.001), and the Hosmer and Lemeshow test was satisfied (*p* = 0.88). The percentage of correct classifications was 93.4%. There was no outlier for which the predicted value of the measured value was above ±3 SD in each modeling.

## 4. Discussion

This study investigated whether the medium-high-risk group in the SBST can be a contributing factor to predict the time and sessions until discharge from MDT and the loss of follow-up without identifying a promising management strategy. Presumably, this is the first study investigating the influence of SBST on MDT.

In the regression model with the dependent variable of the number of days until discharge from MDT, SBST was not a statistically significant contributing factor. This finding indicates that the effectiveness of treatment based on the MDT is not influenced by the SBST, and agrees with a previous finding that the functional status at discharge was not predicted by psychological status [24]. This result reflects that MDT is not a series of exercises, but a classification algorithm to guide identifying a promising management strategy, which is often misunderstood [41]. For example, those who have persistent widespread pain, aggravation with all activities, exaggerated pain behavior, and/or inappropriate beliefs and attitudes about pain are categorized into the subgroup of chronic pain [31], which would be included in the medium-high-risk group in SBST. For this MDT subgroup, any evidence-based approaches, regardless of the names of the approaches, can be incorporated in individualized management by seeking the most responsive strategy for each patient, for example, pain education, graded exposure/activity, coping skills, and other cognitive behavioral therapy techniques [31]. The current finding suggests a need for future studies to compare the MDT, and other approaches as per the effectiveness and cost-effectiveness of each subgroup of risk in the SBST.

In the regression model with the dependent variable of the number of sessions until discharge from MDT, SBST was a statistically significant contributing factor. However, this result should be interpreted with the limitations in this study. The first limitation is the lack of other potential factors to be included in a comprehensive model, for example, (1) therapist–patient alliance, which can be assessed with the working alliance inventory [42]; (2) patient’s attitude toward decision making, which can be assessed with the patient attitude and belief questionnaire [43]; and (3) patient’s exercise adherence, which can be assessed with the exercise adherence rating scale [44]. The second limitation is that the treating-MDT therapist was not masked about the SBST scores and the therapist’s decision-making about the treatment and follow-up might have been influenced by the SBST scores, although the final decision was always made by the patient. It would be assumed that knowing the SBST scores should have enhanced the correlation coefficient to the sessions until discharge of the MDT. SBST was not the primary contributing factor for the number of sessions until discharge from MDT, and the model had a none-very-weak effect size. The contribution of SBST will further decrease when a comprehensive model is investigated with other promising variables. Therefore, it would be prudent not to interpret that SBST was a primary contributing factor for estimating the number of sessions to take until discharge from MDT for now.

Derangement syndrome was a statistically significant contributing factor for time and sessions until discharge from MDT. However, as discussed above, these models excluded all promising variables and had a none-very-weak effect size. Therefore, it would be prudent to not to interpret that the MDT subgroup of derangement syndrome was a primary contributing factor for the effectiveness of treatment and cost in MDT. Such a conservative interpretation agrees with the conclusion of a systematic review, concluding that the importance of derangement syndrome as a prognostic factor is probably overwhelming [45]. Nevertheless, derangement syndrome may be an important treatment effect modifier, and future studies with a validation randomized controlled trial design will be required in order to investigate this hypothesis.

In regression modeling, with the dependent variable being the loss of MDT follow-up, with the mechanically inconclusive subgroup before the fifth session, SBST was a primary contributing factor for the model, where the shortest distance from the patient address to the hospital was also included as a secondary contributing factor. The percentage of correct classifications was 93.4%, indicating that the two factors would be important contributing factors for the loss of follow-up before identifying a promising management strategy. The negative correlation to the shortest distance from the patient address to the hospital may reflect the weight of the patient expectation toward the MDT interventions and classification, suggesting a promising management strategy, where patients who come to the hospital from a long distance are expected to have a greater expectation of the physical therapy session.

Apart from the limitations discussed above, there are other two limitations that should be acknowledged. A limitation is that the data were collected from a single center and by one MDT therapist. The influence of therapists on the treatment effect should be considered [46]. Thus, the generalizability of the results is limited. Another limitation is that patients might have other medical diagnoses that were given in other medical centers that are unshared in the medical record. However, it does not change the conclusion that SBST was not the primary contributing factor, for Question 1 at least.

## 5. Conclusions

SBST status was not a primary contributing factor for time and the number of sessions until discharge from MDT. However, the medium-high-risk group in SBST who resided near the hospital tended to have a loss of MDT follow-up before identifying a promising management strategy.

## Figures and Tables

**Table 1:** Characteristics of the participants.

Variables	Question 1 (*n* = 89)	Question 2 (*n* = 166)
Age, years	61.5 (13.8)	61.6 (14.7)
Gender (*n* of male:*n* of female)	37:52	65:101
The shortest distance from the patient address to the hospital, km	7.6 (6.6)	7.5 (6.6)
Symptom area, 0–11	3.8 (1.8)	3.7 (1.8)
Duration of the current episode of LBP, months	7.7 (17.1)	12.0 (30.7)
The number of medical diagnoses	2.4 (1.7)	2.7 (1.9)
RMDQ, 0–24	8.7 (5.6)	8.8 (5.3)
NRS-LBP, 0–10	4.8 (2.6)	4.9 (2.6)

Abbreviations: LBP—low back pain; RMDQ—Japanese version of the Roland–Morris Disability Questionnaire; NRS-LBP—numerical pain rating scale on LBP at the initial physical therapy session. Values are presented with as the mean (standard deviation), unless specified.

**Table 2:** Results of multiple regression modeling for the number of days until the discharge from the mechanical diagnosis and therapy (MDT).

Model	Unstandardized Coefficients (B)	Standardized Coefficients (β)	*p*-Value (95% Confidence Intervals)
(Constant)	−15.38		0.60 (−73.90–43.14)
MDT subgroup	106.41	0.43	<0.001 (59.24–153.58)

Abbreviations: MDT subgroup, whether the final MDT subgroup was derangement syndrome or not. *R*^2^ = 0.19, analysis of variance *p* < 0.001.

**Table 3:** Results of multiple regression modeling for the number of sessions until the discharge from the mechanical diagnosis and therapy (MDT).

Model	Unstandardized Coefficients (B)	Standardized Coefficients (β)	*p*-Value (95% Confidence Intervals)
(Constant)	−0.41		0.83 (−4.13–3.31)
MDT subgroup	3.85	0.35	0.001 (1.70–6.00)
SBST	1.89	0.22	0.026 (0.23–3.55)

Abbreviations: MDT subgroup, whether the final MDT subgroup was derangement syndrome or not; SBST, whether the Keele STarT Back Screening Tool at the initial session indicated low-risk (total score < 4) or medium−high-risk (total score ≥ 4). *R*^2^ = 0.17, analysis of variance *p* < 0.001.

**Table 4:** Results of logistic regression modeling to estimate whether the patient lost the mechanical diagnosis and therapy follow-up with the mechanically inconclusive subgroup before the fifth session or not.

Model	Partial Regression Coefficient (B)	*p*-Value	Odd Ratio (95% Confidence Intervals)
SBST	1.53	<0.001	4.61 (2.13–10.00)
Distance	−1.59	0.03	0.20 (0.05–0.88)
(Constant)	−4.13	<0.001	

Abbreviation: SBST, whether the Keele STarT Back Screening Tool at the initial session indicated low-risk (total score < 4) or medium−high-risk (total score ≥ 4); Distance, the shortest distance (km) from the patient address to the hospital.
